# Supplementary material for: Variability conceals emerging trend in 100yr projections of UK local hourly rainfall extremes
Source: Nat Commun. 2023 Mar 7;14:1133. doi: 10.1038/s41467-023-36499-9 (PMC9992391; doi:10.1038/s41467-023-36499-9)
Supplement: Supplementary file 1 — Supplementary Information [file 41467_2023_36499_MOESM1_ESM.pdf]

Variability conceals emerging trend in 100yr projections of UK local hourly rainfall extremes

Elizabeth J. Kendon, Erich M. Fischer, Chris J. Short

Supplementary Information

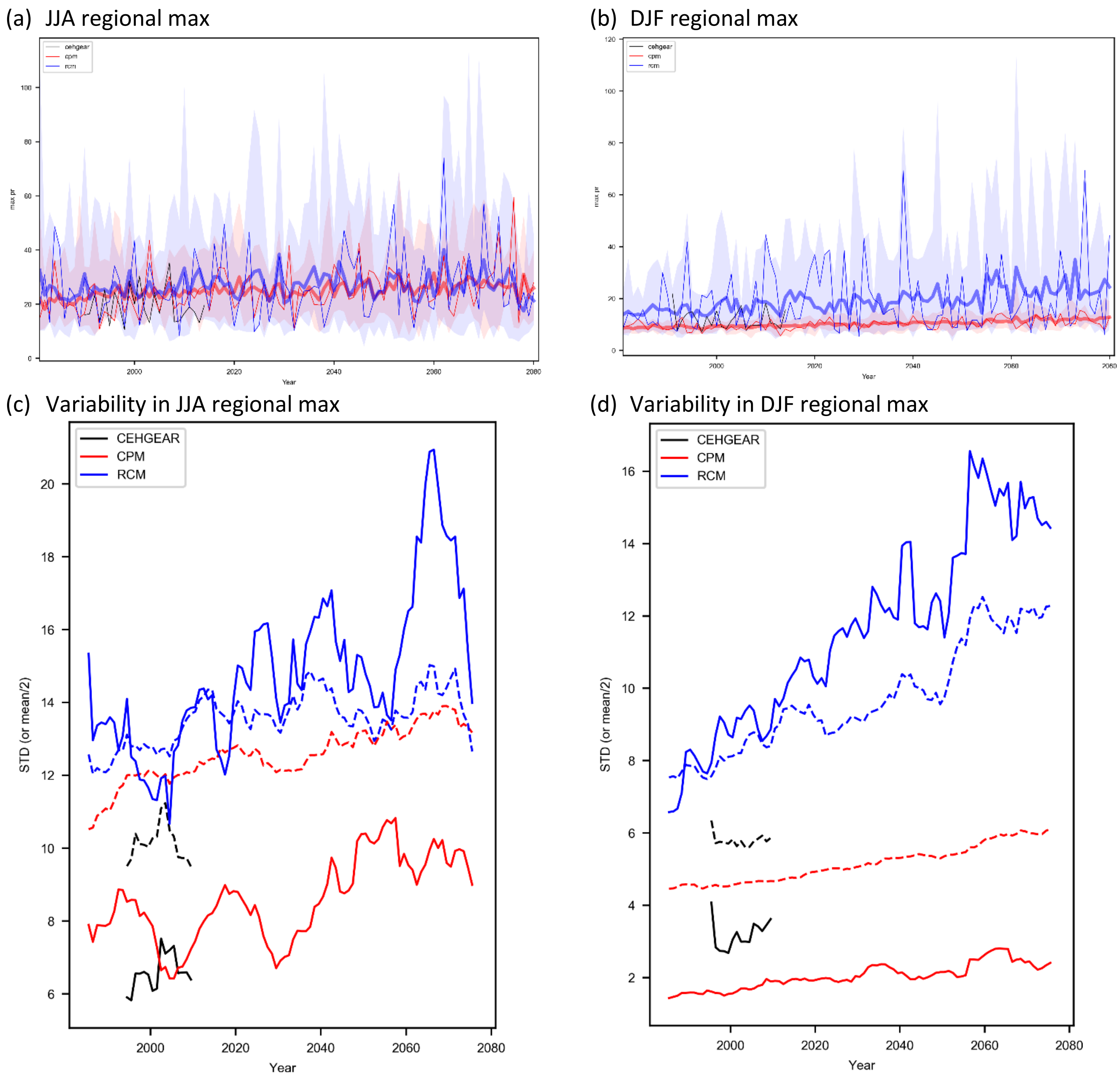

Supp Fig S1: Regional seasonal maximum hourly precipitation (mm/h) for South East England, in summer (June-July-August, JJA) and winter (December-January-February, DJF). Regional seasonal maximum corresponds to the maximum precipitation (mm/h) occurring within the region (considering all 12km grid boxes) in a given season. Results are shown for (a, b) CEHGEAR observations (black, 1990-2014), and in the 2.2km convection-permitting model (CPM, red) and 12km regional climate model (RCM, blue) for the standard member (thin line), ensemble mean (thick line) and ensemble min-max range (shaded) for 1981-2080, for hourly rainfall averaged over 12km grid box. (c, d) Variability in regional seasonal maximum values in running 10-year window, and for the models additionally across the 12 ensemble-members. Solid line shows standard deviation (STD, mm/h) of yearly values, and dashed line the smoothed (multi-member) 10-year running mean (mm/h, divided by 2).

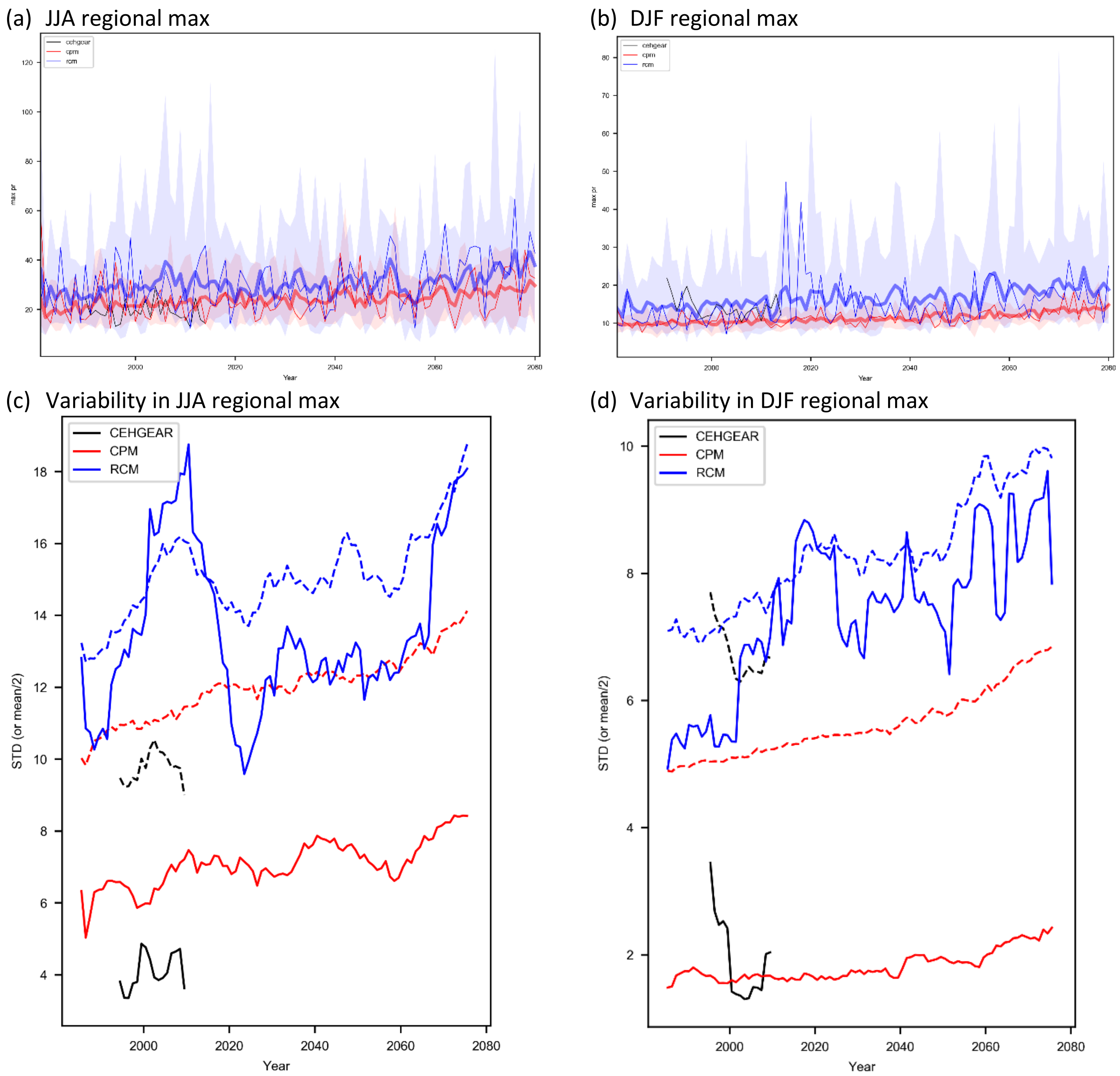

Supp Fig S2: Regional seasonal maximum hourly precipitation (mm/h) for North West England, in summer (June-July-August, JJA) and winter (December-January-February, DJF). Regional seasonal maximum corresponds to the maximum precipitation (mm/h) occurring within the region (considering all 12km grid boxes) in a given season. Results are shown for (a, b) CEHGEAR observations (black, 1990-2014), and in the 2.2km convection-permitting model (CPM, red) and 12km regional climate model (RCM, blue) for the standard member (thin line), ensemble mean (thick line) and ensemble min-max range (shaded) for 1981-2080, for hourly rainfall averaged over 12km grid box. (c, d) Variability in regional seasonal maximum values in running 10-year window, and for the models additionally across the 12 ensemble-members. Solid line shows standard deviation (STD, mm/h) of yearly values, and dashed line the smoothed (multi-member) 10-year running mean (mm/h, divided by 2).

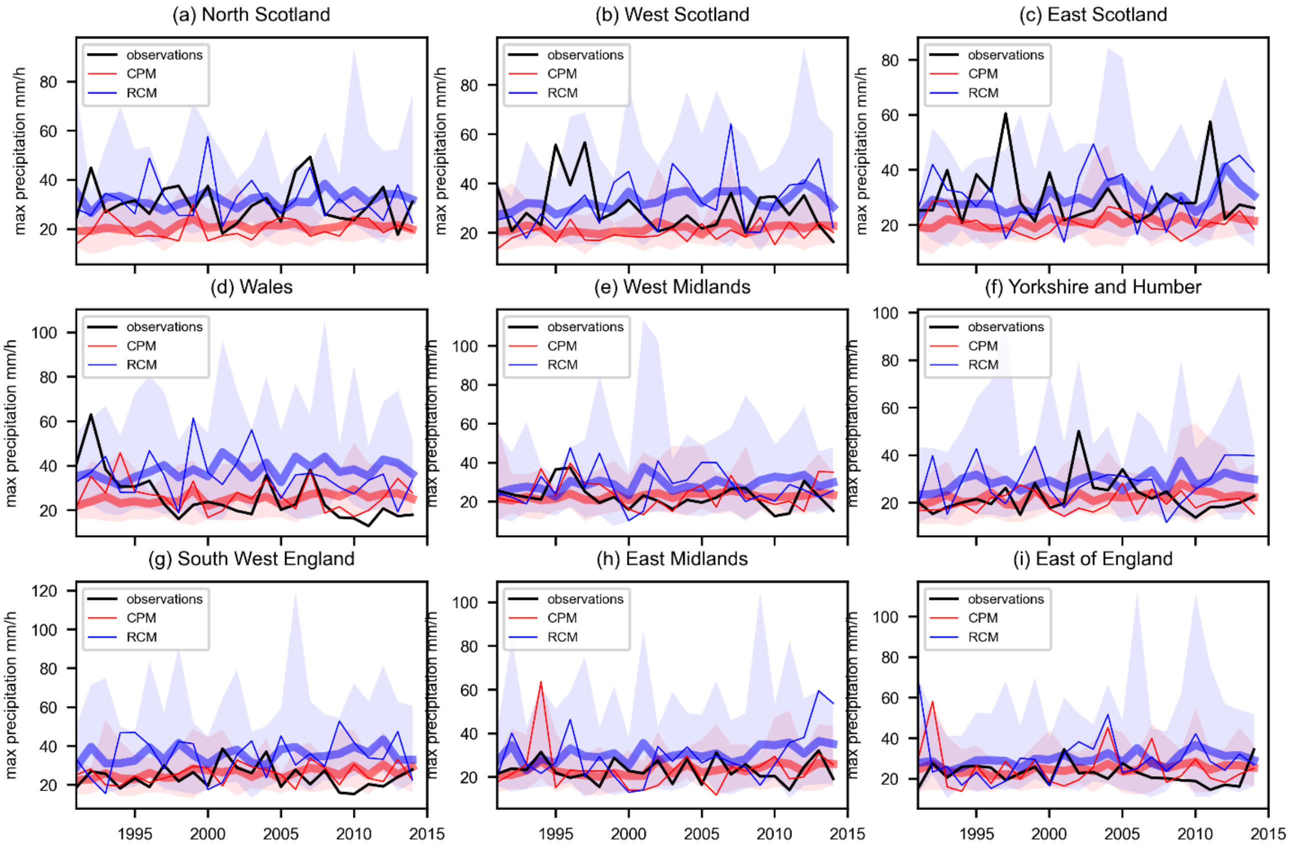

Supp Fig S3: Regional annual maximum hourly precipitation, for regions across the UK. Maximum hourly precipitation (mm/h) occurring within the region (considering all 12km grid boxes) in a given year, for 1991-2014. Results are shown for CEHGEAR observations (black), and the convection-permitting model (CPM, red) and regional climate model (RCM, blue), for the standard member (thin line), ensemble mean (thick line) and ensemble min-max range (shaded). Regions are as shown in Fig 4.

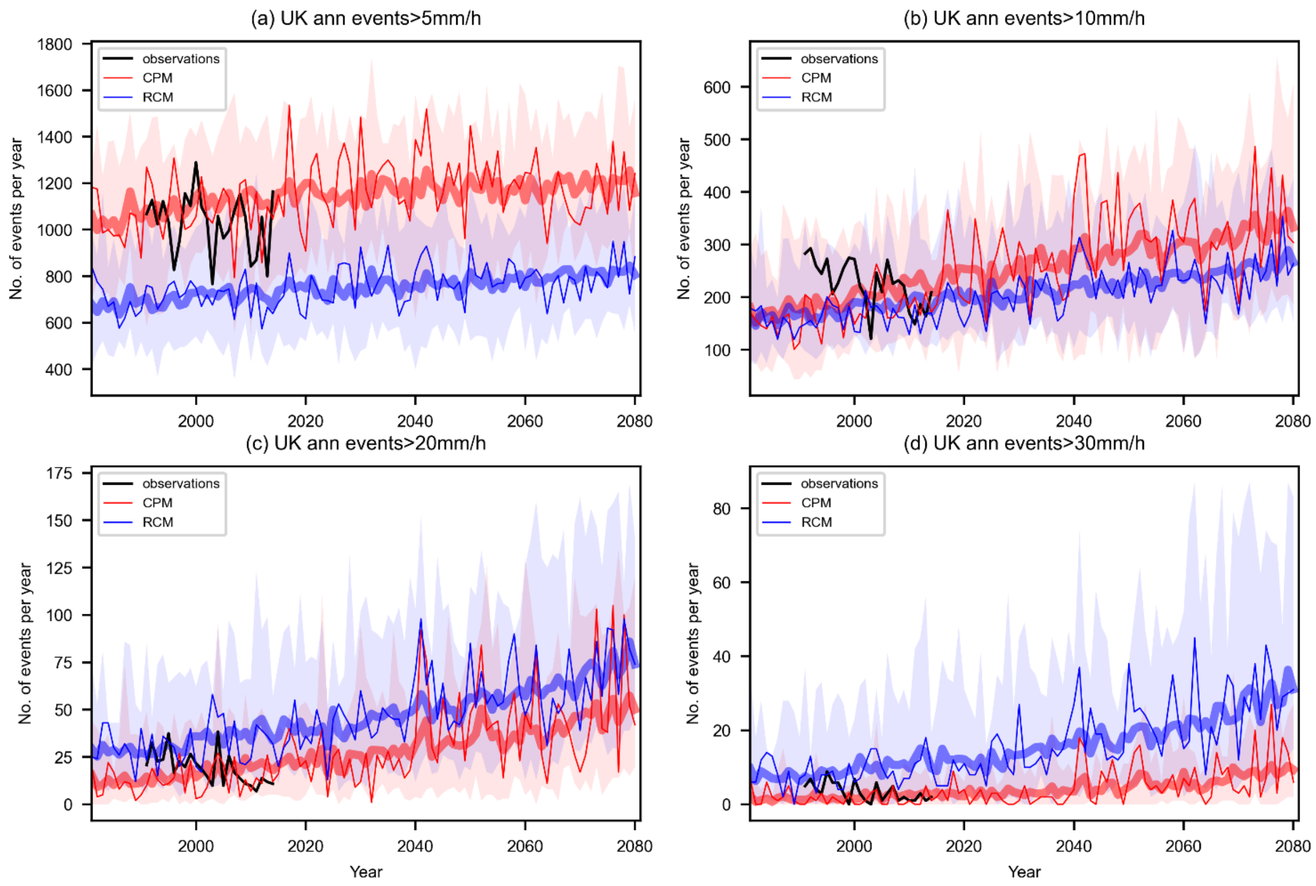

Supp Fig S4: Frequency of precipitation events exceeding a range of high thresholds. Number of events per year across the UK (UK ann events) exceeding (a) 5mm/h, (b) 10mm/h (c) 20mm/h and (d) 30mm/h, for precipitation at 12km scale. Results are shown for CEHGEAR observations (black, 1991-2014), and the convection-permitting model (CPM, red) and regional climate model (RCM, blue) for the standard member (thin line), ensemble mean (thick line) and ensemble min-max range (shaded) for 1981-2080. Threshold exceedances occurring within a UK subregion on the same day are considered part of a single event.

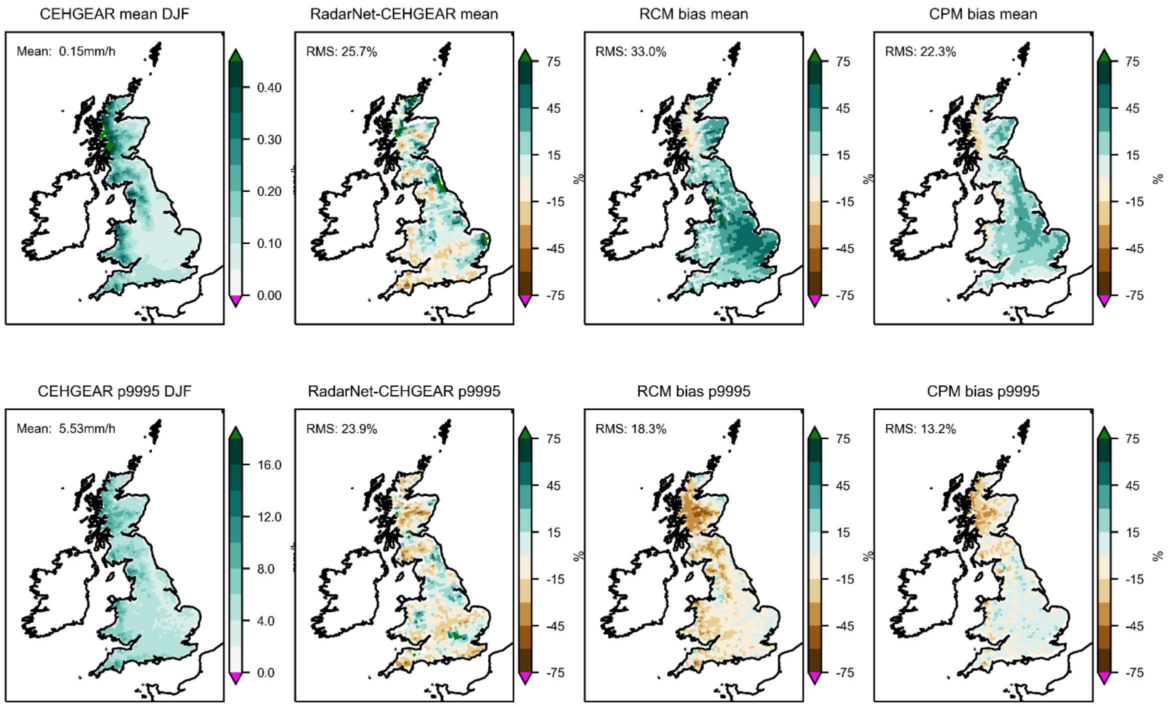

Supp Fig S5. Model performance in winter. (top) Seasonal mean precipitation and (bottom) 99.95th percentile of hourly precipitation (mm/h), in December-January-February DJF, for (left) CEHGEAR (1990-2014) and differences (%) with respect to the (centre left) radar (2003-2017), (centre right) regional climate model (RCM) and (right) convection-permitting model (CPM) ensemble mean for the baseline climate (1981-2000). The UK average values (in mm/h) and root mean square errors (RMS, in %) are indicated.

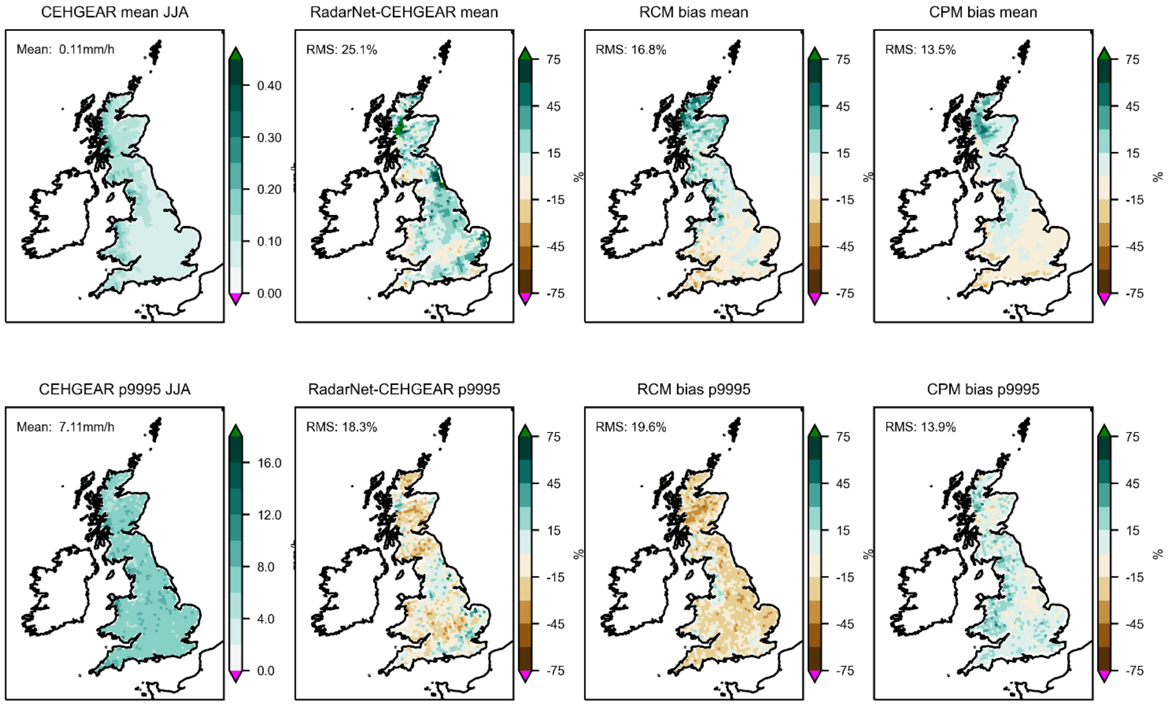

Supp Fig S6. Model performance in summer. (top) Seasonal mean precipitation and (bottom) 99.95th percentile of hourly precipitation (mm/h), in June-July-August JJA, for (left) CEHGEAR (1990-2014) and differences (%) with respect to the (centre left) radar (2003-2017), (centre right) regional climate model (RCM) and (right) convection-permitting model (CPM) ensemble mean for the baseline climate (1981-2000). The UK average values (in mm/h) and root mean square errors (RMS, in %) are indicated.

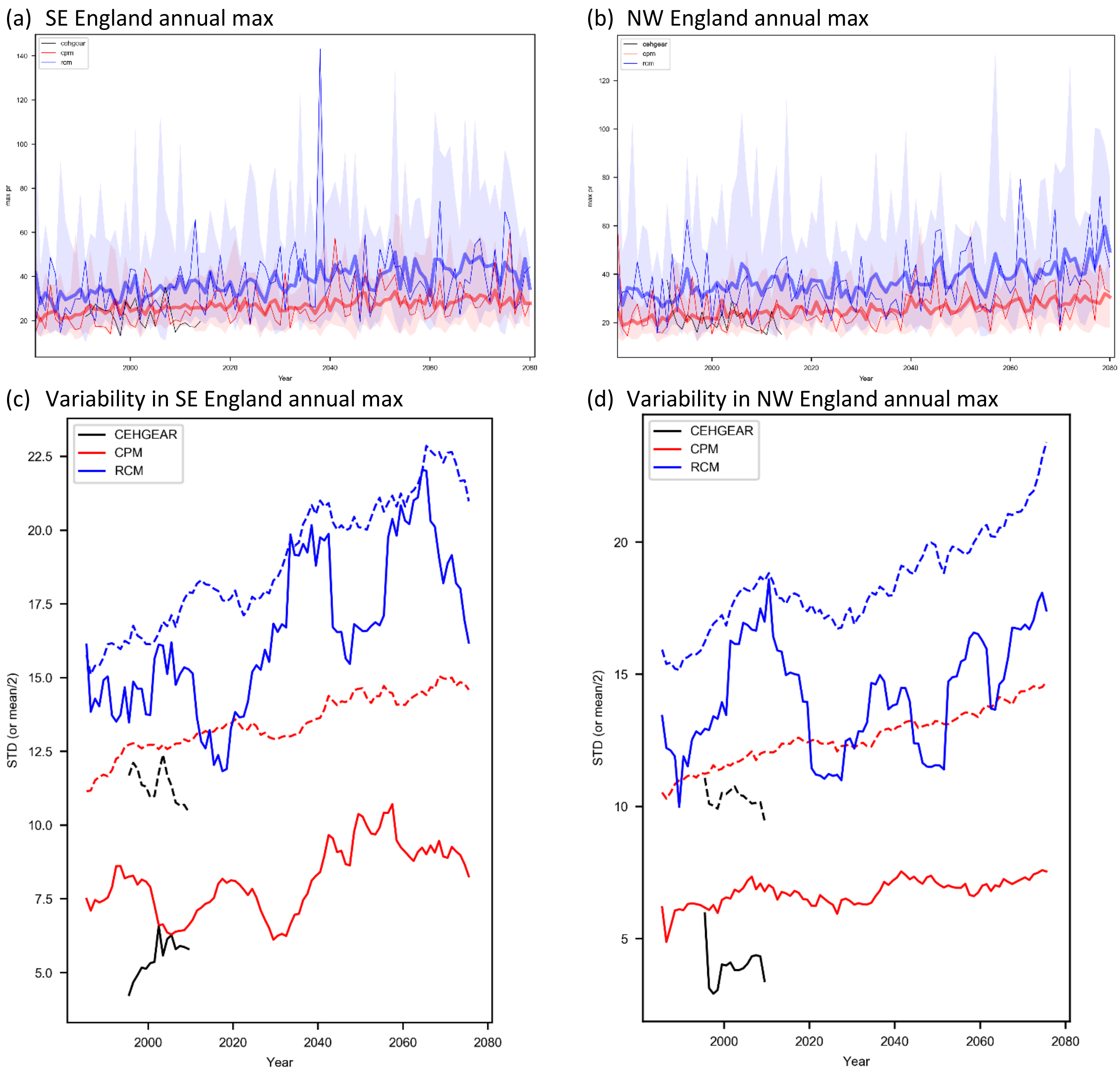

Supp Fig S7: Regional annual maximum hourly precipitation (mm/h) for (left) South East (SE) England and (right) North West (NW) England. (a, b) The maximum precipitation (mm/h) occurring within the region (considering all 12km grid boxes) in a given year for CEHGEAR observations (black, 1991-2014), and in the convection-permitting model (CPM, red) and regional climate model (RCM, blue) for the standard member (thin line), ensemble mean (thick line) and ensemble min-max range (shaded) for 1981-2080, for hourly rainfall averaged over 12km grid box. (c, d) Variability in regional annual maximum values in running 10-year window, and for the models additionally across the 12 ensemble-members. Solid line shows standard deviation (STD, mm/h) of yearly values, and dashed line the smoothed (multi-member) 10-year running mean (mm/h, divided by 2).

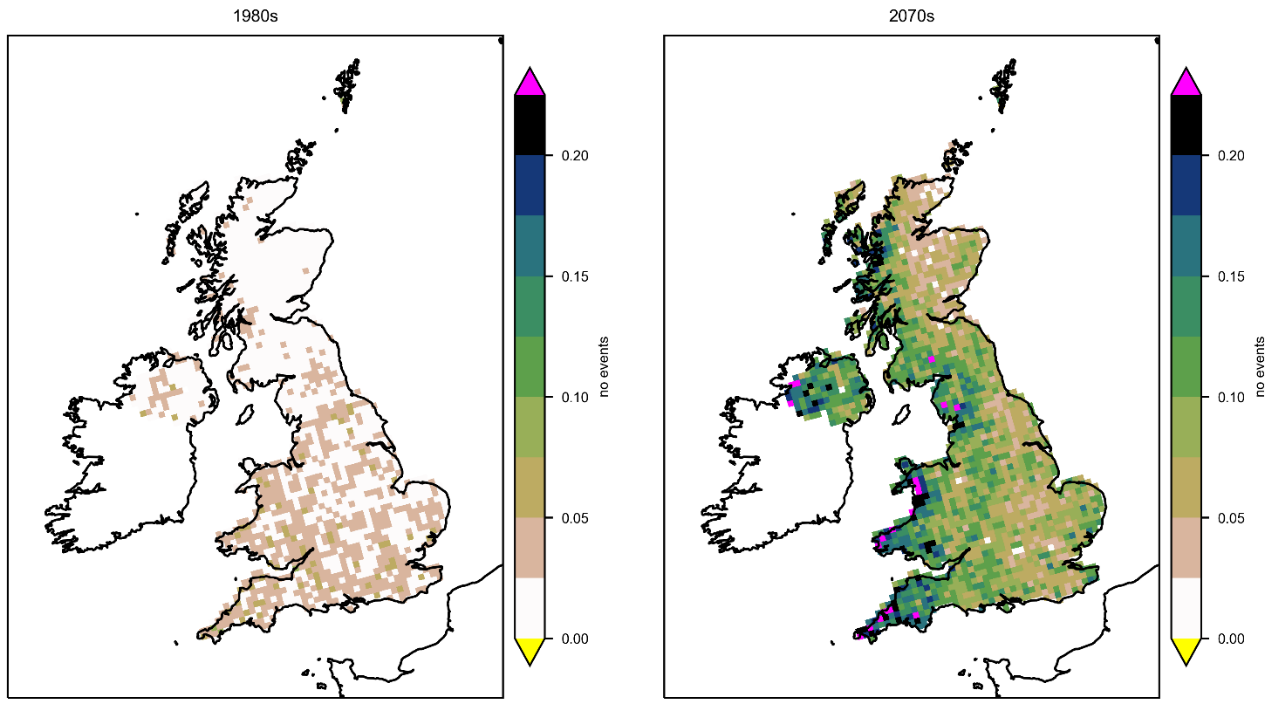

Supp Fig S8: Average number of events per year exceeding 20mm/h locally in the 1980s and 2070s, in the convection-permitting model (CPM). Values correspond to the multi-member 10-year mean number of events, for hourly rainfall averaged over a 12km grid box.

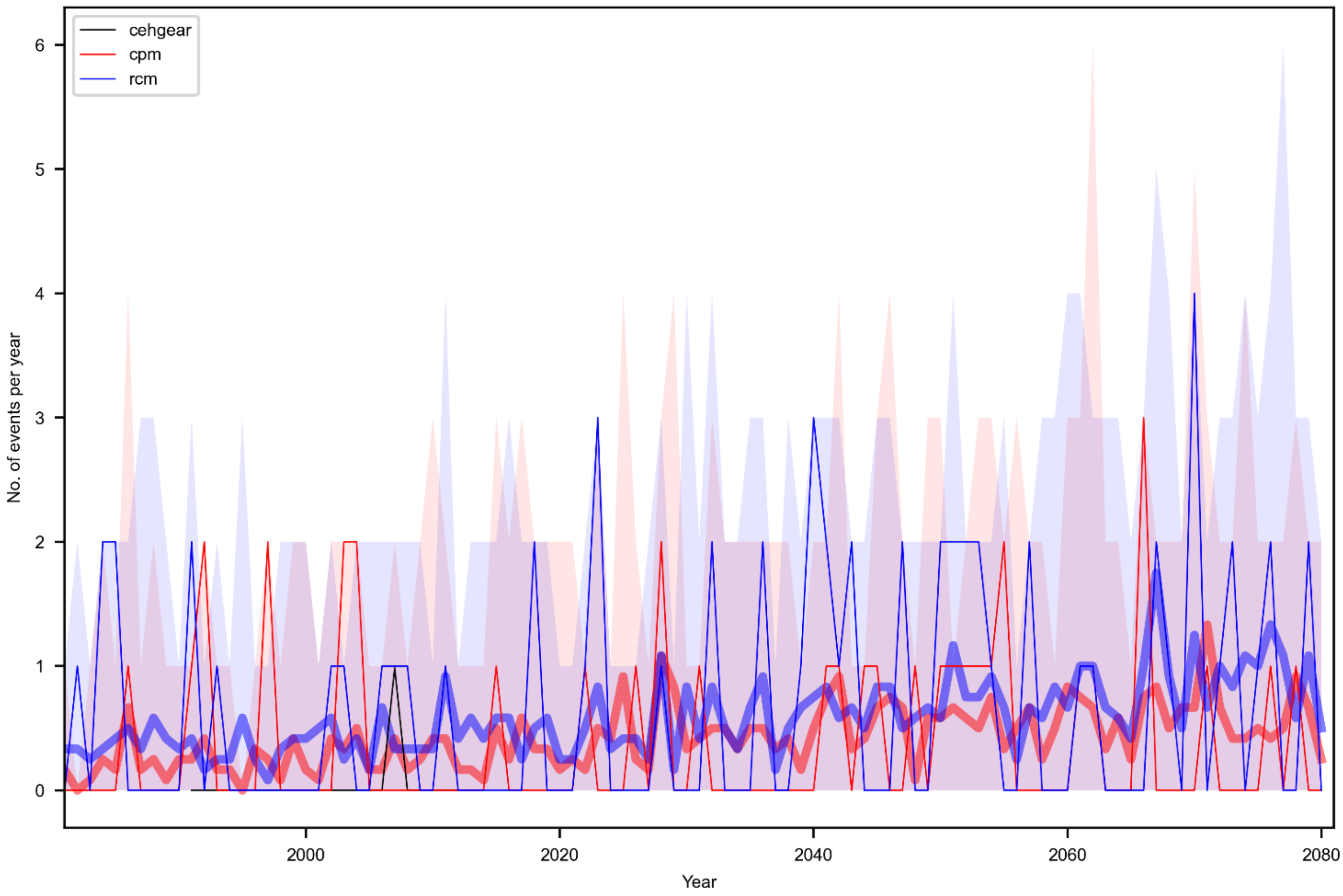

Supp Fig S9: Number of events per year across London exceeding 20mm/h, for hourly rainfall averaged over 12km grid box. Threshold exceedances occurring within London on the same day are considered part of a single event. Results are shown for the observations (1991-2014), and the convection-permitting model (CPM) and regional climate model (RCM) ensembles (1981-2080). The thick line shows the ensemble mean, thin line the standard member, and the shaded region the ensemble min-max range for each year.

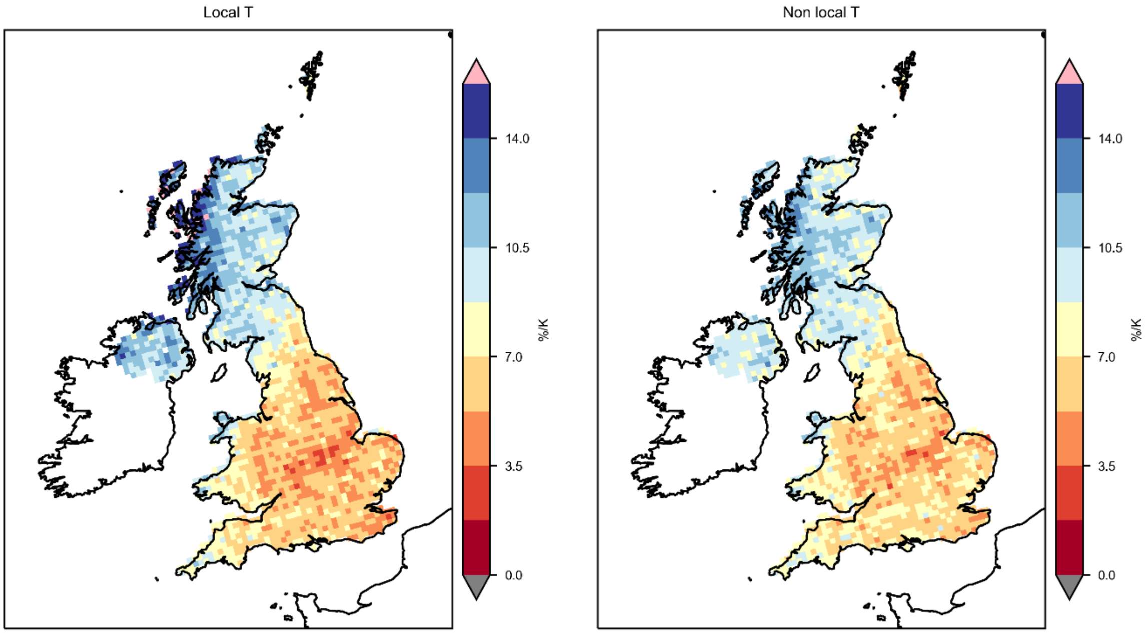

Supp Fig S10: Scaling coefficients (%/K) for underlying climate change signal in local annual maximum hourly precipitation in the convection-permitting model (CPM). Values correspond to the gradient of a linear trend line fitted to smoothed annual maximum precipitation versus annual mean temperature, (left) using local temperature at the 12km scale and (right) using UK-average temperature, expressed as % increase in precipitation per K temperature increase. The smoothed data consists of the multi-member 10-year running mean.

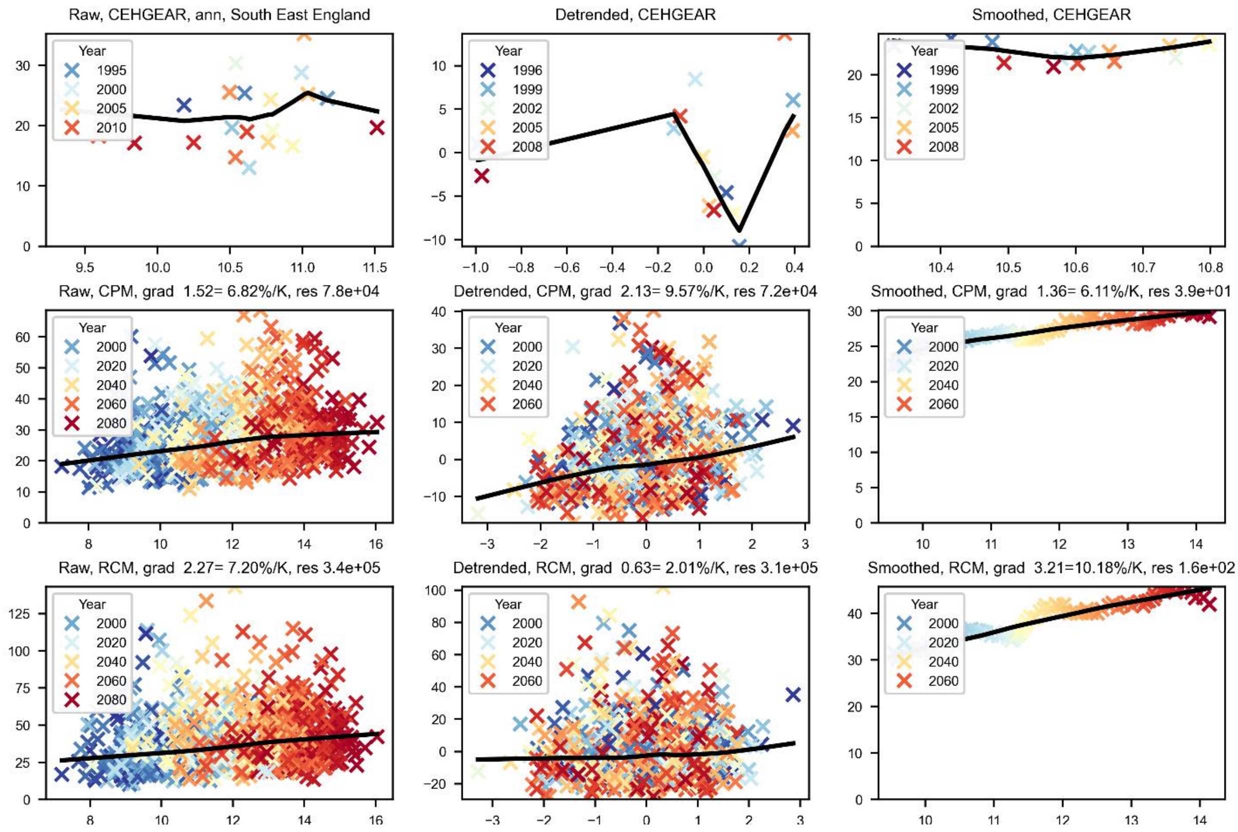

Supp Fig S11: Temperature scaling of regional annual maximum hourly precipitation for South East England, for CEHGEAR observations, convection-permitting model (CPM) and regional climate model (RCM) ensembles. Shown is the highest annual (ann) maximum hourly 12km precipitation (mm/h) across grid points plotted against the regional average annual temperature (°C) per year, for raw data, detrended data and smoothed data. For raw model data, there are 12 points for each year, corresponding to the 12 ensemble members. The smoothed data corresponds to the multi-member 10-year running mean. Detrended data is calculated by subtracting the multi-member 10-year running mean from the yearly value for each ensemble member, for both the regional maximum precipitation and the regional mean temperature. A lowess regression has been fitted to the data (black line), and colours correspond to the year. Titles give the gradient (grad) of and residual (res) from a linear trend line, with the gradient converted to % increase in precipitation per K temperature increase.

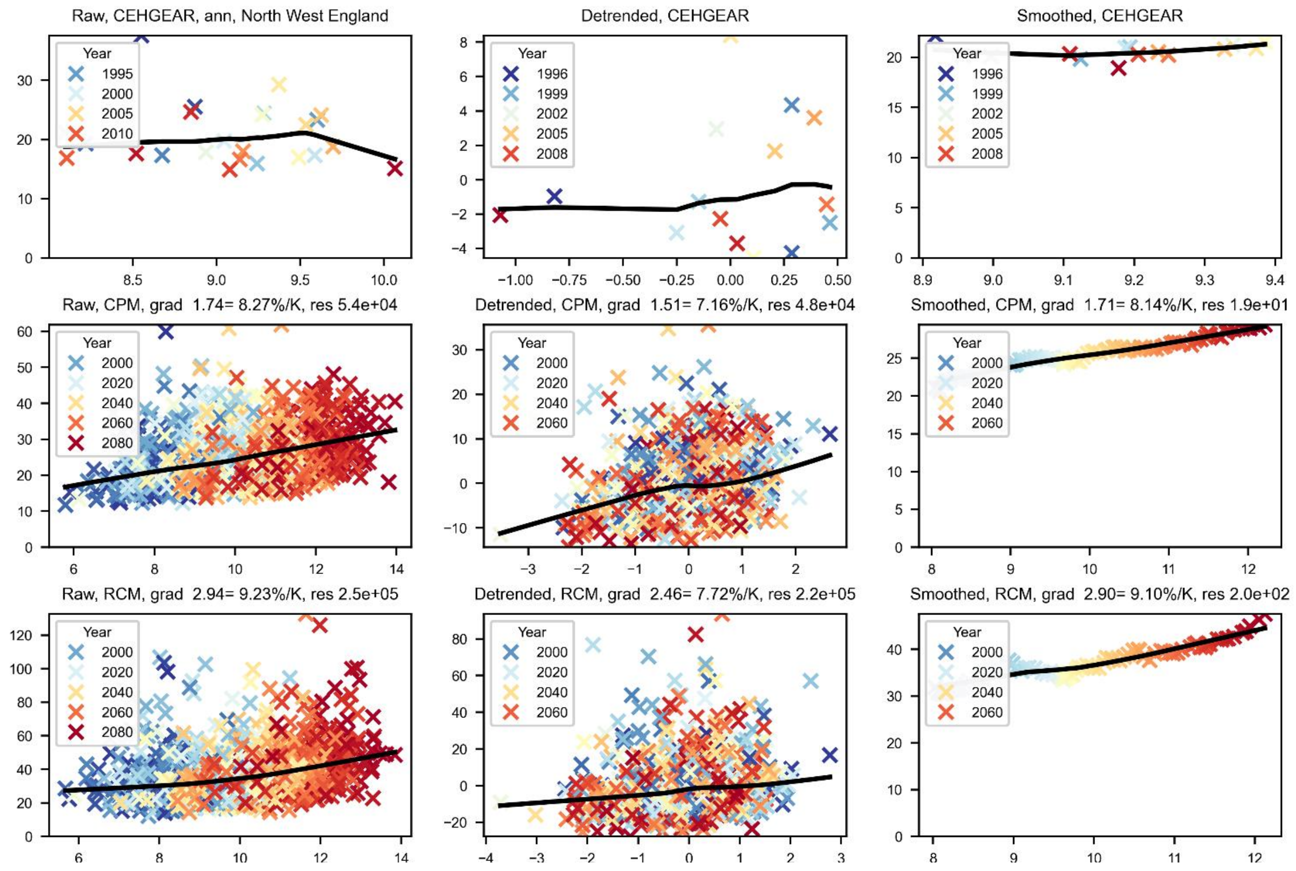

Supp Fig S12: Temperature scaling of regional annual maximum hourly for North West England, for CEHGEAR observations, convection-permitting model (CPM) and regional climate model (RCM) ensembles. Shown is the highest annual (ann) maximum hourly 12km precipitation (mm/h) across grid points plotted against the regional average annual temperature (°C) per year, for raw data, detrended data and smoothed data. For raw model data, there are 12 points for each year, corresponding to the 12 ensemble members. The smoothed data corresponds to the multi-member 10-year running mean. Detrended data is calculated by subtracting the multi-member 10-year running mean from the yearly value for each ensemble member, for both the regional maximum precipitation and the regional mean temperature. A lowess regression has been fitted to the data (black line), and colours correspond to the year. Titles give the gradient (grad) of and residual (res) from a linear trend line, with the gradient converted to % increase in precipitation per K temperature increase.

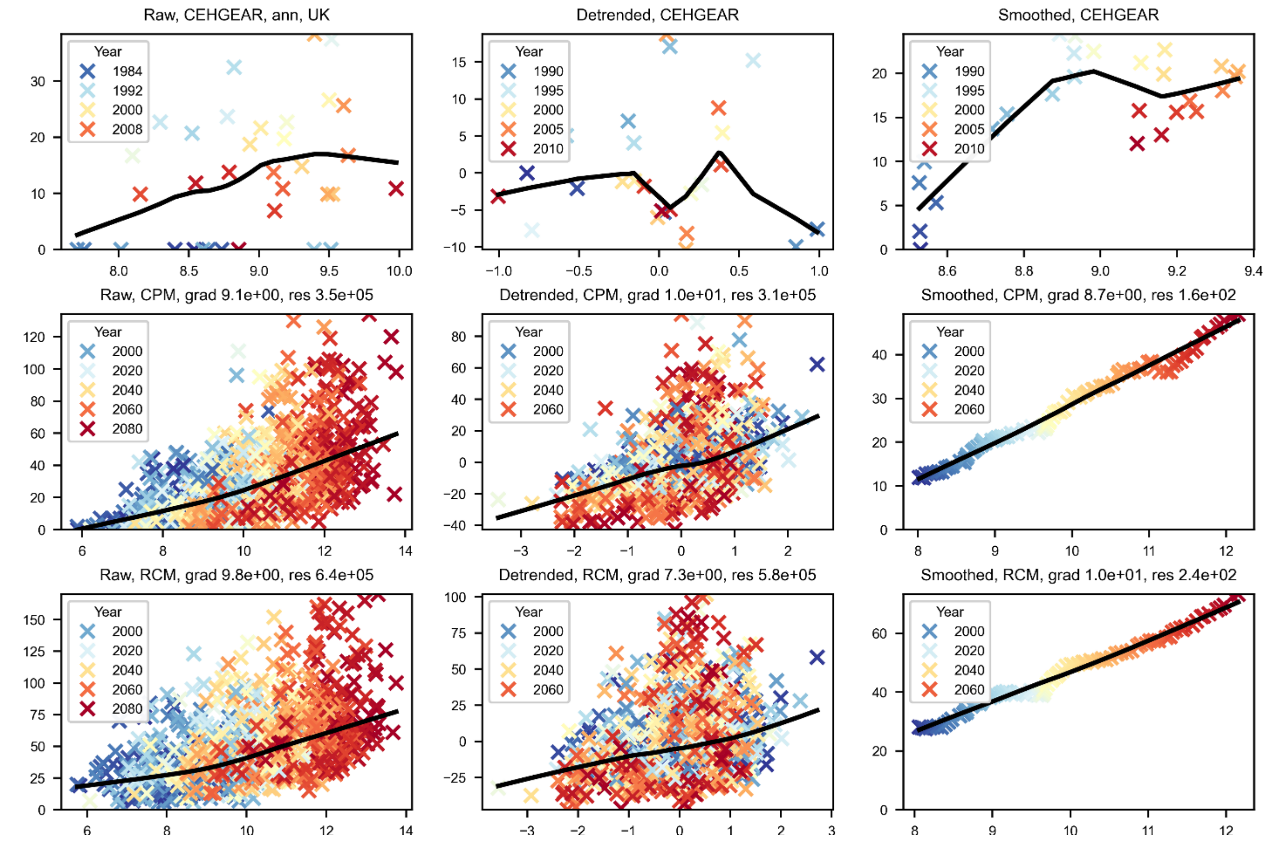

Supp Fig S13: Temperature scaling of number of events exceeding 20mm/h, for CEHGEAR observations, convection-permitting model (CPM) and regional climate model (RCM) ensembles. Shown is the number of events across the UK per year, plotted against the UK average annual temperature (°C), for raw data, detrended data and smoothed data. Threshold exceedances occurring within a UK subregion on the same day are considered part of a single event. For raw model data, there are 12 points for each year, corresponding to the 12 ensemble members. The smoothed data corresponds to the multi-member 10-year running mean. Detrended data is calculated by subtracting the multi-member 10-year running mean from the yearly value for each ensemble member, for both the number of precipitation events and the UK mean temperature. A lowess regression has been fitted to the data (black line), and colours correspond to the year. Titles give the gradient (grad) of and residual (res) from a linear trend line.

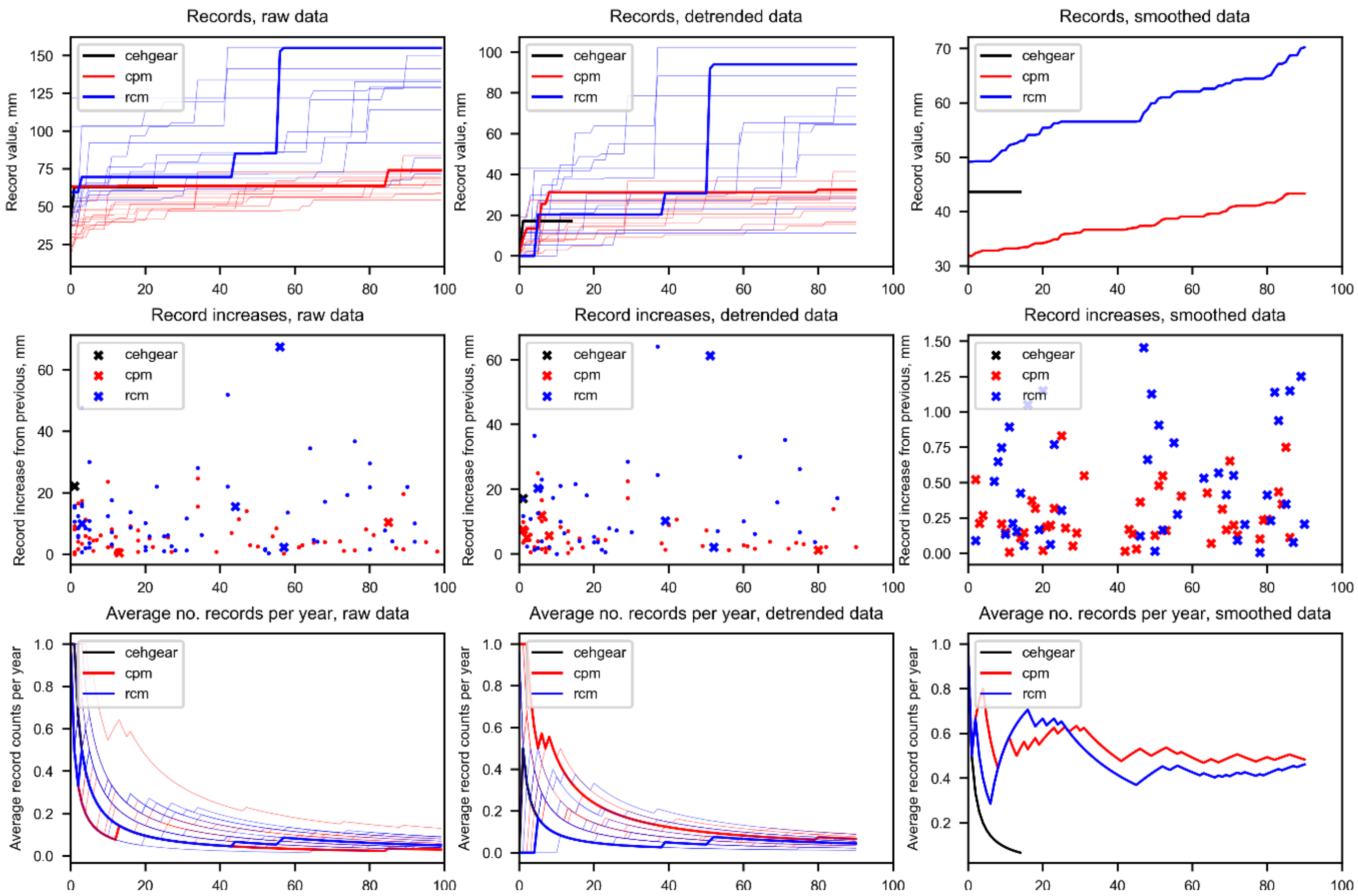

Supp Fig S14: Exceedance of UK records as a function of year from the start of the data series. (top) Highest value (mm/h) of annual maximum hourly precipitation recorded at any grid point across the UK since the start of the dataseries, (middle) increase in record from the previous values set, and (bottom) average number of records per year from the start of the dataseries. Results are shown for (left) raw yearly timeseries of annual maximum hourly precipitation, (centre) detrended data where the multi-member 10-year running mean of annual maximum values has been removed and (right) the smoothed multi-member 10-year running mean timeseries. The dataseries for CEHGEAR observations (black) is for 1991-2014, whilst for the convection-permitting model (CPM, red) and regional climate model (RCM, blue) is for 1981-2080. Hourly precipitation has been averaged onto a common 12km grid before calculation of regional maximum values. For the models, the standard member is shown as bold, or a cross in centre panels.

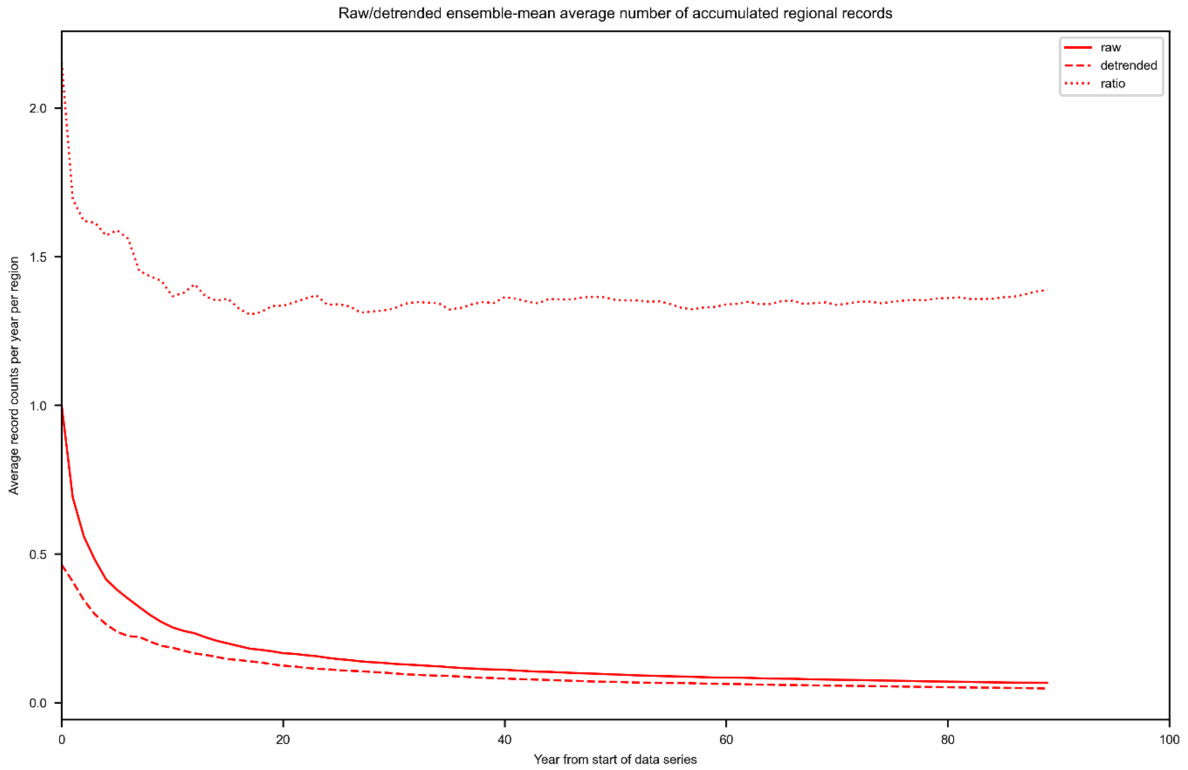

Supp Fig S15: Exceedance of regional records of local hourly precipitation across the UK in the convection-permitting model (CPM). Shown is the ensemble mean average number of regional records per year, summed across all UK regions, plotted against years from the start of the data series. Results are shown for raw data (solid), detrended data (dashed) and their ratio (dotted). Detrended data is where the multi-member 10-year running mean of annual maximum values has been removed. This is as Fig 8(d) except for regional records accumulated across the UK, rather than UK-wide records. UK regions are defined in Fig 4.

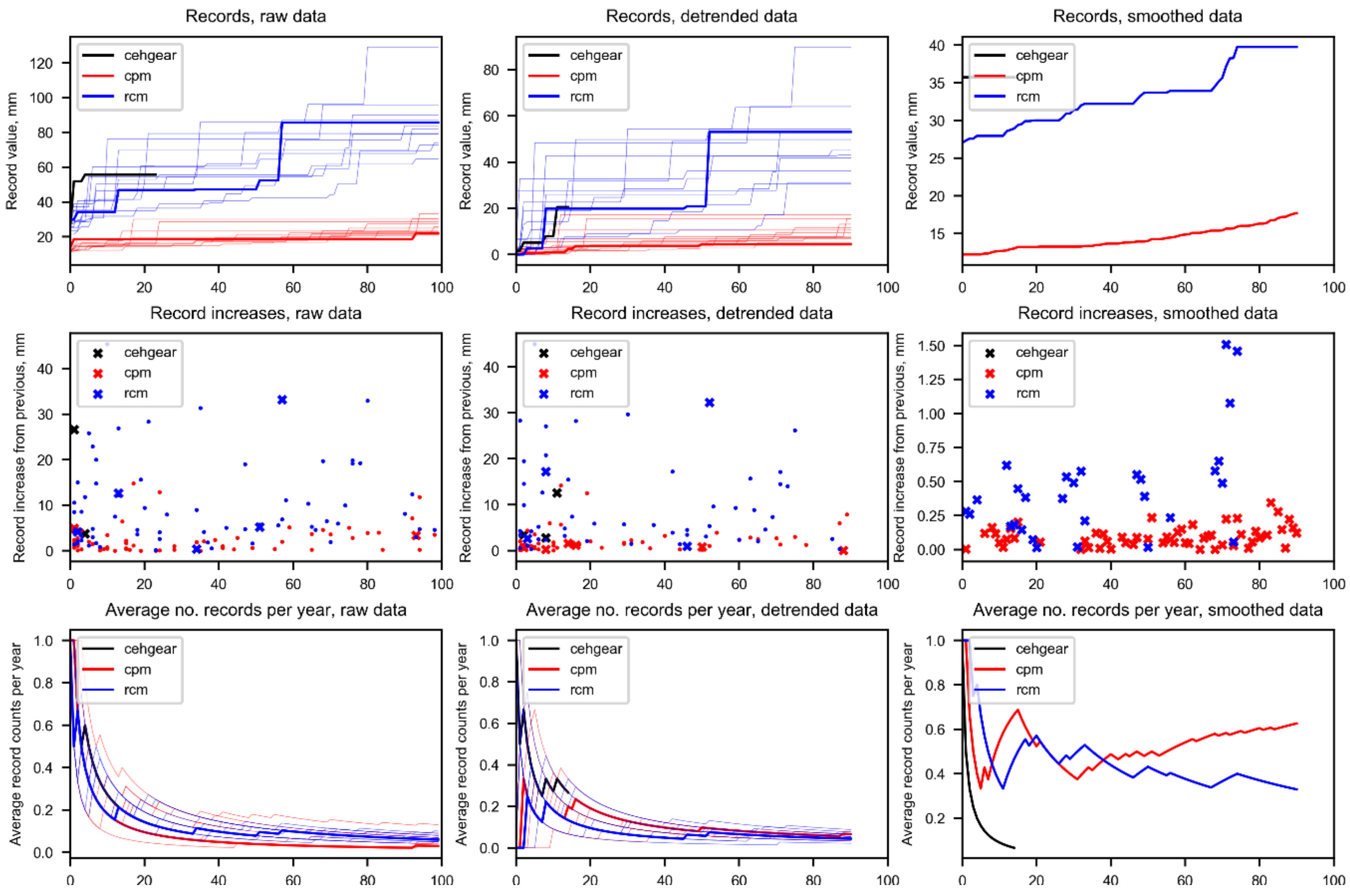

Supp Fig S16: Exceedance of UK winter records in local hourly precipitation as a function of year from the start of the data series. As Supp Fig S14, but for winter season only. (top) Highest value (mm/h) of winter maximum hourly precipitation recorded at any grid point across the UK since the start of the dataseries, (middle) increase in record from the previous values set, and (bottom) average number of records per year from the start of the dataseries. Results are shown for (left) raw yearly timeseries of winter maximum hourly precipitation, (centre) detrended data where the multi-member 10-year running mean of winter maximum values has been removed and (right) the smoothed multi-member 10-year running mean timeseries. The dataseries for CEHGEAR observations (black) is for 1991-2014, whilst for the convection-permitting model (CPM, red) and regional climate model (RCM, blue) is for 1981-2080. Hourly precipitation has been averaged onto a common 12km grid before calculation of regional maximum values. For the models, the standard member is shown as bold, or a cross in centre panels.

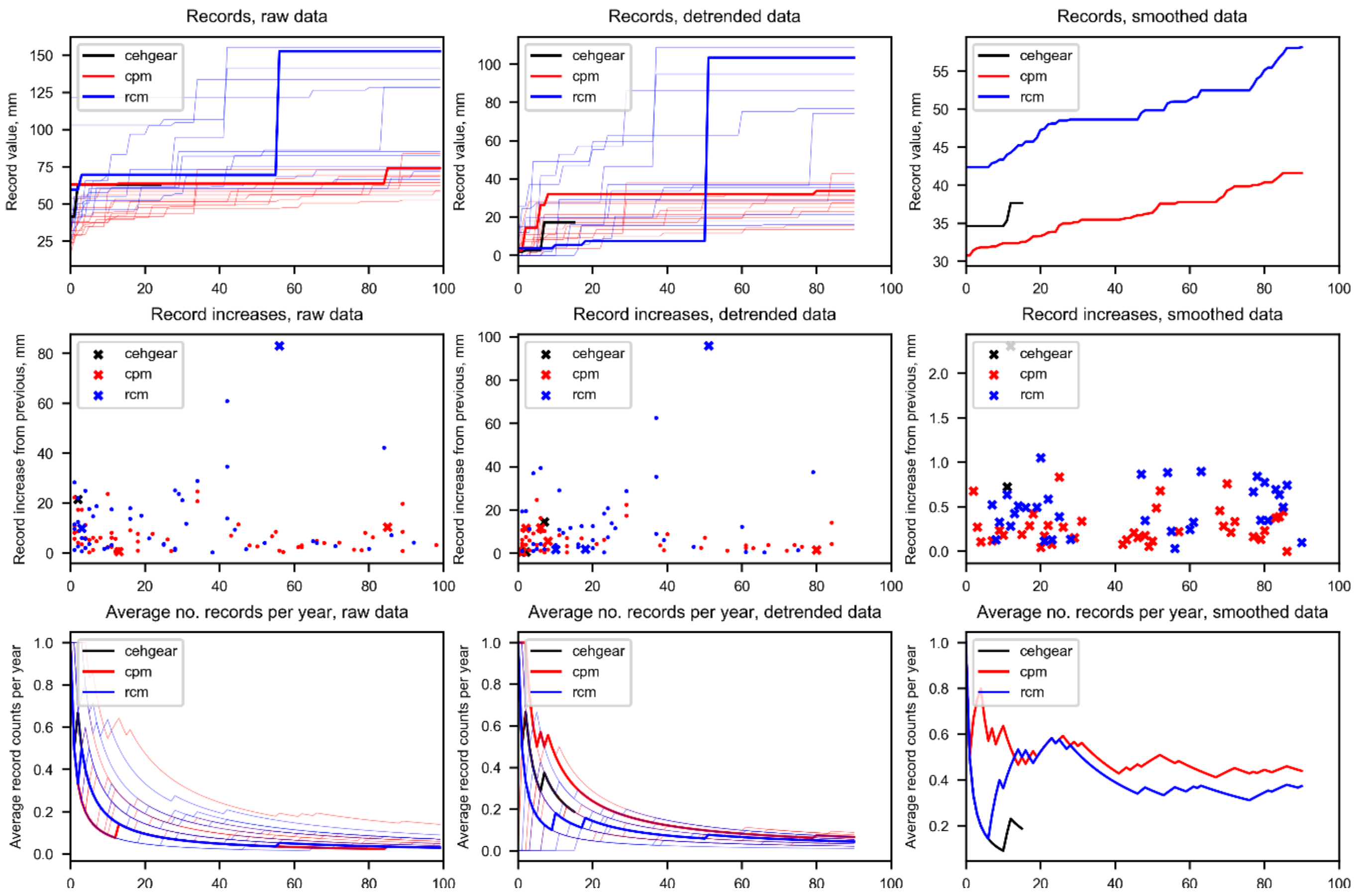

Supp Fig S17: Exceedance of UK summer records in local hourly precipitation as a function of year from the start of the data series. As Supp Fig S16 but for summer.

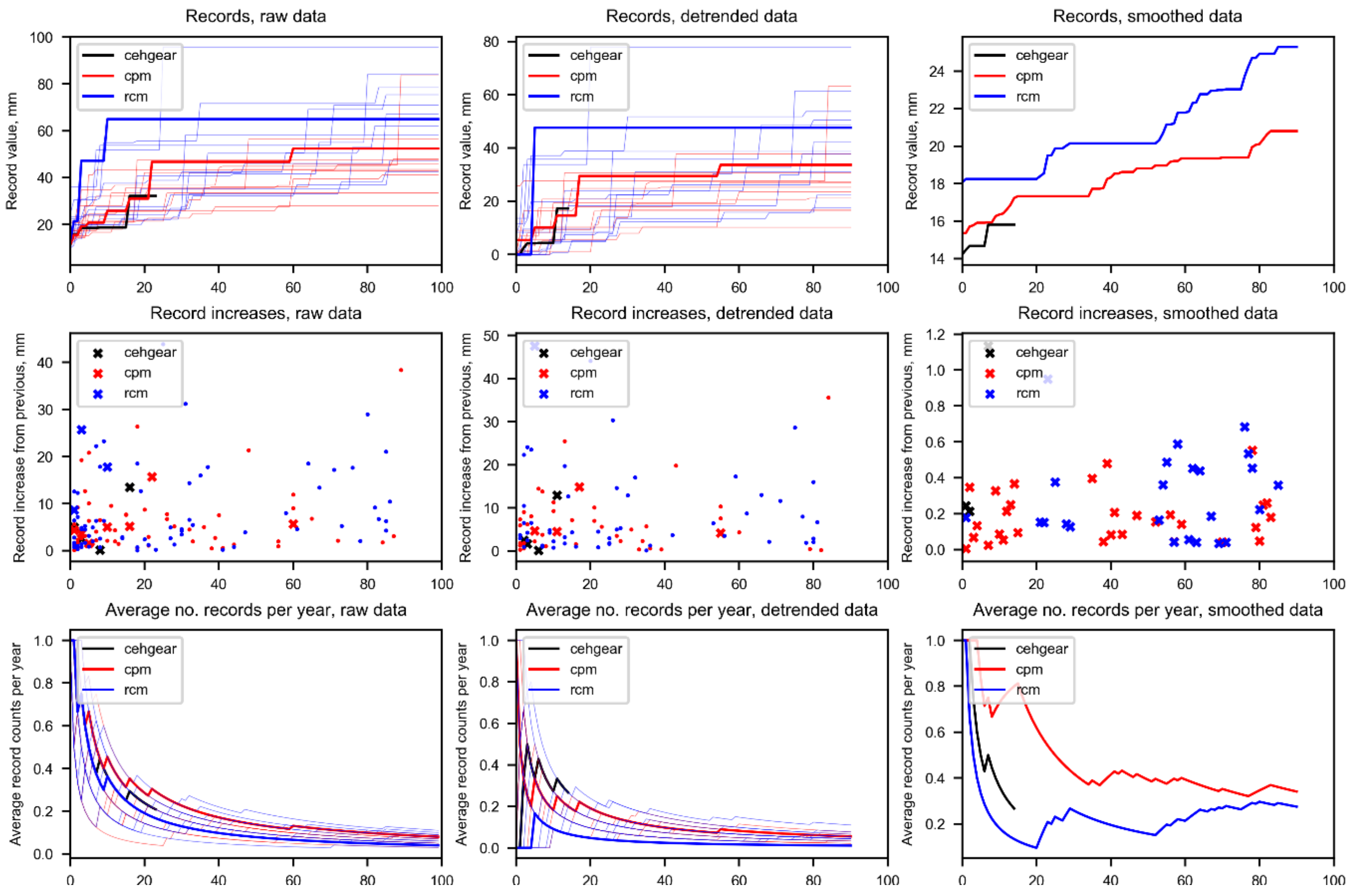

Supp Fig S18: Exceedance of London records in local hourly precipitation as a function of year from the start of the data series. As Supp Fig S14, but for London only. (top) Highest value (mm/h) of annual maximum hourly precipitation recorded at any grid point across London since the start of the dataseries, (middle) increase in record from the previous values set, and (bottom) average number of records per year from the start of the dataseries. Results are shown for (left) raw yearly timeseries of annual maximum hourly precipitation, (centre) detrended data where the multi-member 10-year running mean of annual maximum values has been removed and (right) the smoothed multi-member 10-year running mean timeseries. The dataseries for CEHGEAR observations (black) is for 1991-2014, whilst for the convection-permitting model (CPM, red) and regional climate model (RCM, blue) is for 1981-2080. Hourly precipitation has been averaged onto a common 12km grid before calculation of regional maximum values. For the models, the standard member is shown as bold, or a cross in centre panels.

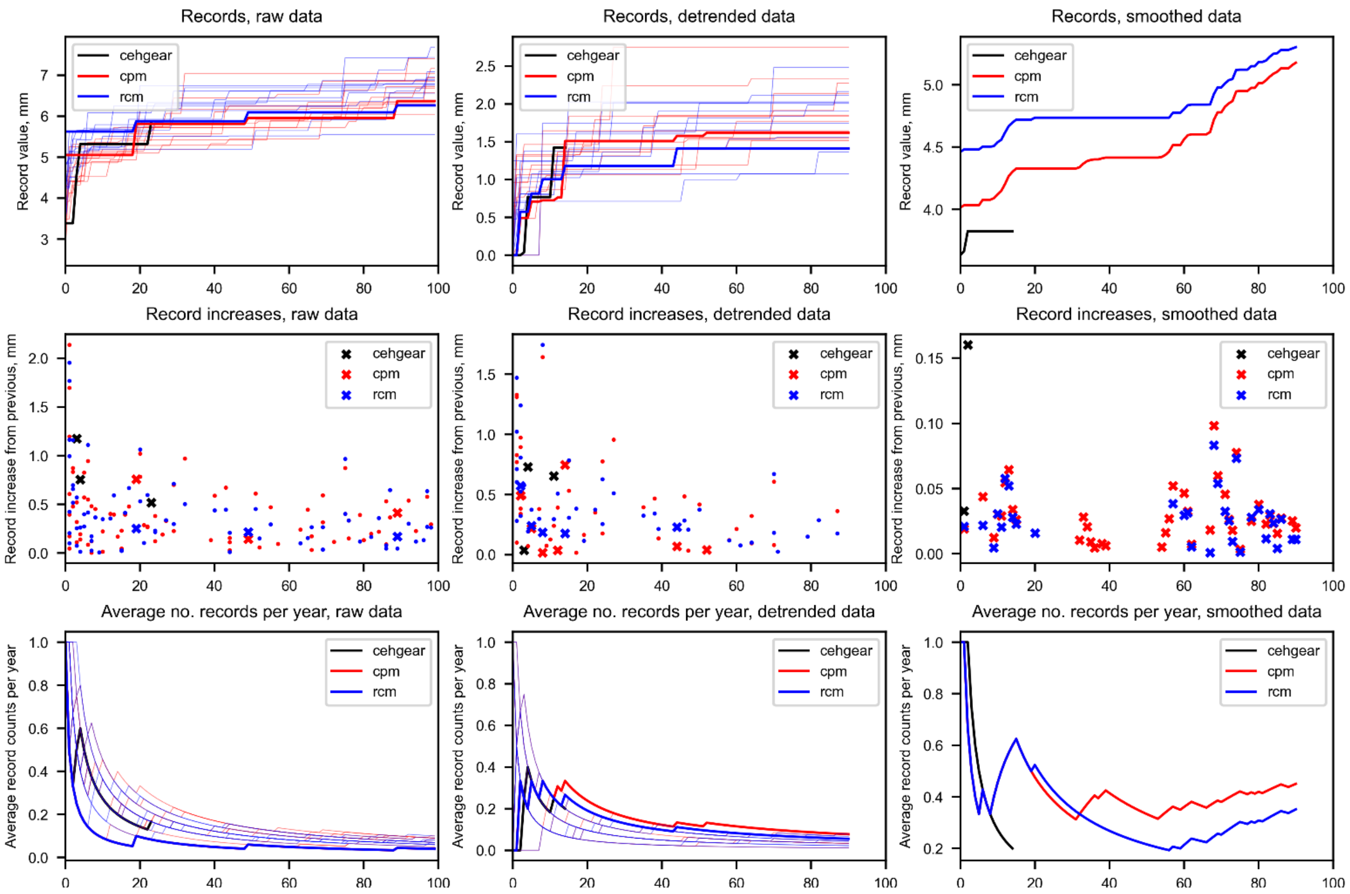

Supp Fig S19: Exceedance of UK-average winter-mean rainfall records as a function of year from the start of the data series. (top) Highest value (mm/d) of UK-average winter-mean precipitation recorded since the start of the dataseries, (middle) increase in record from the previous values set, and (bottom) average number of records per year from the start of the dataseries. Results are shown for (left) raw yearly timeseries of winter average precipitation, (centre) detrended data where the multi-member 10-year running mean of winter mean values has been removed and (right) the smoothed multi-member 10-year running mean timeseries. The dataseries for CEHGEAR observations (black) is for 1991-2014, whilst for the convection-permitting model (CPM, red) and regional climate model (RCM, blue) is for 1981-2080. For the models, the standard member is shown as bold, or a cross in centre panels.

Supplementary Tables

Supp Table S1: Average number of events per year exceeding 20mm/h, 10mm/h or the present-day 99.99th percentile of hourly precipitation in the 1980s and 2070s, and their future change, for regions of the UK. Values correspond to the multi-member 10-year mean number of events, for the convection-permitting model and in brackets regional climate model, CPM (RCM). The 99.99th percentile threshold is calculated by pooling all 12km grid boxes across the UK and all hours in the 20-year baseline period (1981-2000). Threshold exceedances occurring within a UK subregion on the same day are considered part of a single event.

Threshold	Region	No events per year in 1980s	No events per year in 2070s	Future/control
20mm/h	**UK**	**11.9 (28.1)**	**49.2 (73.3)**	**4.14 (2.61)**
	North Scotland	0.54 (3.15)	5.30 (9.20)	9.78 (2.93)
	West Scotland	0.67 (2.52)	4.42 (7.80)	6.62 (3.09)
	East Scotland	0.53 (1.83)	3.97 (7.00)	7.57 (3.82)
	Northern Ireland	0.53 (1.31)	3.21 (4.30)	6.11 (3.29)
	North East England	0.45 (0.78)	2.05 (2.95)	4.56 (3.77)
	North West England	0.78 (2.66)	3.95 (6.63)	5.10 (2.50)
	Yorkshire and Humber	0.77 (1.49)	2.77 (4.13)	3.61 (2.77)
	Wales	1.54 (3.57)	5.80 (9.01)	3.76 (2.53)
	West Midlands	0.86 (1.38)	2.27 (2.87)	2.65 (2.08)
	East Midlands	0.93 (1.62)	2.64 (3.59)	2.86 (2.21)
	East of England	1.06 (1.79)	3.35 (3.88)	3.17 (2.16)
	South West England	1.19 (3.21)	5.32 (6.51)	2.79 (2.03)
	South East England	1.16 (2.40)	3.57 (4.48)	3.09 (1.87)
	London	0.21 (0.38)	0.61 (0.92)	2.92 (2.39)
10mm/h	**UK**	**173 (162)**	**338 (263)**	**1.95 (1.62)**
	North Scotland	16.0 (25.1)	36.9 (37.8)	2.31 (1.50)
	West Scotland	15.8 (18.3)	35.3 (30.1)	2.24 (1.64)
	East Scotland	11.2 (12.4)	26.4 (24.6)	2.36 (1.99)
	Northern Ireland	11.0 (8.11)	20.8 (14.8)	1.89 (1.82)
	North East England	6.74 (5.42)	15.4 (1.04)	2.28 (1.92)
	North West England	15.7 (13.9)	33.0 (25.1)	2.10 (1.80)
	Yorkshire and Humber	9.72 (7.56)	19.9 (14.1)	2.05 (1.87)
	Wales	21.8 (20.3)	39.3 (31.5)	1.81 (1.55)
	West Midlands	8.90 (6.00)	15.5 (9.43)	1.74 (1.57)
	East Midlands	9.31 (7.17)	16.6 (11.5)	1.79 (1.61)
	East of England	11.4 (7.98)	18.1 (12.3)	1.59 (1.54)
	South West England	19.7 (16.4)	33.0 (22.6)	1.67 (1.38)
	South East England	13.1 (11.2)	22.4 (15.3)	1.72 (1.37)
	London	2.72 (2.21)	5.47 (3.77)	2.02 (1.71)
99.99th percentile of hourly precipitation (9.28 mm/h	**UK**	**219 (189)**	**399 (296)**	**1.82 (1.57)**
	North Scotland	20.5 (29.7)	43.7 (42.7)	2.13 (1.44)
	West Scotland	20.7 (21.6)	41.8 (33.9)	2.02 (1.57)
	East Scotland	15.1 (14.4)	31.3 (27.4)	2.08 (1.90)
	Northern Ireland	13.9 (9.36)	24.6 (16.7)	1.77 (1.78)

in CPM and 9.35 mm/h in RCM)	North East England	8.57 (6.52)	18.3 (11.8)	2.14 (1.81)
	North West England	20.1 (16.4)	39.2 (28.8)	1.95 (1.75)
	Yorkshire and Humber	12.3 (8.87)	24.0 (15.9)	1.94 (1.79)
	Wales	27.4 (23.8)	46.4 (35.7)	1.69 (1.50)
	West Midlands	11.0 (6.83)	18.3 (10.6)	1.67 (1.56)
	East Midlands	11.5 (8.14)	19.6 (12.8)	1.71 (1.58)
	East of England	13.8 (9.07)	20.8 (13.7)	1.50 (1.51)
	South West England	24.5 (18.7)	38.6 (25.2)	1.57 (1.35)
	South East England	16.2 (12.6)	26.0 (17.0)	1.60 (1.35)
	London	3.60 (2.49)	6.67 (4.32)	1.85 (1.73)

Supp Table S2: Temperature scaling of regional maximum hourly precipitation. Values correspond to the gradient of a linear trend line fitted to the yearly or seasonal maximum precipitation versus mean temperature, expressed as % increase in precipitation per K temperature increase. Results are shown for the raw data, detrended data and smoothed underlying climate change signal, for December-January-February (DJF), June-July-August (JJA) and annual. Results are shown for the convection permitting model (CPM) and regional climate model (RCM) as CPM/RCM. York + Hum = Yorkshire + Humber region.

	**DJF**			**JJA**			**Annual**		
	**Raw**	**Detrended**	**Smoothed**	**Raw**	**Detrended**	**Smoothed**	**Raw**	**Detrended**	**Smoothed**
UK	8.16/10.07	3.99/7.59	12.49/14.10	7.43/5.10	8.69/2.17	6.73/6.39	8.60/7.37	9.12/1.96	8.53/9.74
London	7.88/12.80	6.05/8.03	9.84/19.87	3.38/1.97	4.76/1.70	3.20/2.66	6.56/8.80	7.85/8.03	6.62/9.91
E Scotland	5.72/1.69	4.45/0.39	6.68/4.32	11.89/10.96	13.78/8.56	10.56/12.14	13.51/10.83	13.70/5.28	13.39/14.21
N Scotland	6.56/1.52	4.90/1.72	7.74/0.88	12.95/12.49	13.44/10.19	13.34/13.88	14.03/10.55	13.79/5.99	14.77/12.98
W Scotland	7.59/6.10	4.89/5.65	10.83/6.64	10.45/10.12	12.46/9.97	9.13/9.74	12.11/10.41	13.61/6.53	11.60/12.26
N Ireland	6.87/8.20	5.30/6.61	8.92/10.78	11.83/11.81	14.26/10.92	10.05/11.68	14.32/14.25	16.01/9.44	13.13/16.78
E Midlands	8.25/11.21	6.05/8.03	10.69/15.30	5.89/3.18	7.62/0.74	5.11/3.46	7.40/6.06	8.87/1.62	6.90/6.99
E England	8.43/11.13	6.40/8.00	10.52/15.22	5.89/3.43	7.58/-1.71	5.30/5.91	7.83/7.97	8.36/2.75	7.88/10.39
NE England	6.27/2.02	5.56/0.83	7.25/3.57	8.77/9.31	11.95/6.60	6.90/10.05	9.89/12.19	11.19/5.52	9.08/14.30
NW England	7.54/10.30	4.73/9.39	10.82/12.66	7.39/5.76	8.27/5.89	6.08/4.63	8.27/9.23	7.16/7.72	8.14/9.10
SE England	7.96/14.64	5.41/12.01	10.68/18.99	4.78/0.56	6.63/-0.83	3.96/2.08	6.82/7.20	9.57/2.01	6.11/10.18
SW England	7.66/13.09	3.99/10.81	12.00/16.12	4.58/2.28	6.46/0.36	3.94/3.16	7.64/9.10	9.53/5.11	6.98/11.17
Wales	8.42/11.11	3.87/8.38	13.63/14.11	6.39/4.53	8.56/4.31	5.06/4.52	8.62/9.13	10.04/5.79	7.89/10.32
W Midlands	6.33/12.92	4.65/10.41	8.45/18.26	4.48/1.75	5.95/2.13	3.31/1.37	6.14/4.95	7.53/2.53	5.19/6.12
York + Hum	6.60/8.47	4.71/6.93	8.62/11.22	7.06/5.24	10.45/2.03	5.23/6.09	7.83/8.01	10.67/0.66	6.69/10.72
